# Association of Life’s Crucial 9 Score With Liver Fibrosis and Mortality in U.S. Adults With MASLD: Evidence From NHANES and the Mediating Role of Systemic Inflammation

**DOI:** 10.1155/mi/2700500

**Published:** 2026-06-19

**Authors:** Zhuo Chen, Lin Shi, Junjie Lu, Jing Su

**Affiliations:** ^1^ Department of Gastroenterology, Xuzhou Central Hospital, Southeast University, Xuzhou, Jiangsu, China, seu.edu.cn; ^2^ Department of Neurology, Xuzhou Central Hospital, Southeast University, Xuzhou, Jiangsu, China, seu.edu.cn

**Keywords:** all-cause mortality, Life Crucial 9, liver fibrosis, mediation analysis, metabolic dysfunction-associated steatotic liver disease

## Abstract

**Background:**

Metabolic dysfunction‐associated steatotic liver disease (MASLD) is increasingly prevalent. Life’s Crucial 9 (LC9) combines cardiometabolic and mental health metrics linked to MASLD pathogenesis, but its association with liver fibrosis and prognosis in MASLD remains unknown.

**Methods:**

Using National Health and Nutrition Examination Survey (NHANES) data, we performed a prospective cohort analysis for mortality (2005–2016, *n* = 2524) and a cross‐sectional analysis for liver fibrosis (2017–2018, *n* = 1277). Significant fibrosis was defined as liver stiffness measurement (LSM) ≥8.0 kPa by vibration‐controlled transient elastography. Systemic inflammation was assessed using the systemic immune‐inflammation index (SII) and pan‐immune‐inflammation value (PIV). Multivariable regression and mediation analyses were conducted.

**Results:**

Higher LC9 scores were associated with lower liver stiffness (*β* = −0.07, 95% CI: −0.12 to −0.01). Each 1‐point increase in LC9 reduced fibrosis risk by 5% (OR = 0.95), with the highest quartile showing the lowest risk (OR = 0.21). Over a median follow‐up of 98 months, each 1‐point LC9 increase was associated with a 3% reduction in all‐cause mortality (HR = 0.97). Lower systemic inflammation partially mediated the LC9‐mortality relationship (SII: 5%, PIV: 6.3%).

**Conclusion:**

Higher LC9 scores are associated with reduced liver fibrosis and improved survival in MASLD, with systemic inflammation partially mediating the mortality association.

## 1. Introduction

Metabolic dysfunction‐associated steatotic liver disease (MASLD), which was once referred to as nonalcoholic fatty liver disease (NAFLD), has become a major global health issue, with an increasing prevalence in parallel with the rising rates of obesity, type 2 diabetes, and metabolic syndrome [1]. Unlike the prior term NAFLD, the MASLD nomenclature does not require the exclusion of other liver disease causes (e.g., viral hepatitis) and better reflects the disease’s close association with metabolic syndrome, offering a more practical approach in clinical practice [2]. MASLD encompasses a spectrum of liver conditions, ranging from simple steatosis to advanced liver fibrosis, cirrhosis, and ultimately, hepatocellular carcinoma [2]. Notably, approximately 10% of MASLD patients may progress to cirrhosis within 20 years, highlighting the risk of long‐term complications and liver‐related mortality [3]. Early detection and monitoring of liver fibrosis are essential for risk stratification and timely intervention, yet noninvasive biomarkers and effective risk models remain limited.

Life’s Crucial 9 (LC9) is a multidimensional health risk score developed to assess cardiovascular health [4]. Expanding on the American Heart Association’s Life’s Essential 8 (LE8), LC9 incorporates a mental health dimension alongside traditional factors such as diet, physical activity, weight, blood lipids, blood pressure, smoking, and fasting blood glucose [4–6]. While LE8 focuses on cardiovascular health metrics, LC9 integrates a mental health component. This addition is clinically relevant because depression is highly prevalent in MASLD patients and has been associated with worse metabolic outcomes and adherence to lifestyle interventions [7]. Recent studies have demonstrated that LC9 has been shown to predict adverse cardiovascular outcomes [4], but its application in liver disease, particularly in the context of MASLD, remains unclear.

Chronic low‐grade inflammation plays an important role in the development of MASLD and its advancement to liver fibrosis [8, 9]. Systemic inflammation is associated with hepatic steatosis, liver injury, and fibrogenesis [2]. We, therefore, evaluated inflammatory response, we utilize the systemic immune‐inflammation index (SII) and the pan‐immune‐inflammation value (PIV), both of which reflect chronic low‐grade inflammation and have been linked to metabolic diseases such as fatty liver [10, 11]. Biologically, hyperglycemia and insulin resistance activate de novo lipogenesis and promote pro‐fibrotic cytokine production (e.g., TGF‐β). Obesity‐related adipose tissue dysfunction releases free fatty acids and inflammatory adipokines, exacerbating hepatic inflammation, and stellate cell activation [12]. Together, these pathways provide a mechanistic link between the cardiometabolic components of LC9 and liver fibrogenesis.

In this study, we used data from the National Health and Nutrition Examination Survey (NHANES), which is a nationally representative, cross‐sectional survey designed to assess the health and nutritional status of U.S. adults and children. It uniquely combines in‐home interviews and physical examinations, collecting data on demographics, medical history, and laboratory parameters (www.cdc.gov/nchs/nhanes). Specifically, we evaluated the association between LC9 and prognosis during the 2005–2016 NHANES cycles, and in the 2017–2018 cycle, which included liver ultrasound data, we assessed the relationship between LC9 and liver fibrosis. The availability of liver ultrasound in the 2017–2018 data allows for a more accurate evaluation of liver fibrosis, enhancing the precision of our study.

Given the overlap in metabolic risk factors between cardiovascular disease and MASLD, we hypothesize that (1) higher LC9 scores are associated with lower risk of liver fibrosis and all‐cause mortality in MASLD patients and (2) systemic inflammation, as measured by SII and PIV, partially mediates the protective effect of LC9 on mortality. Accordingly, this study aims to (a) evaluate the association between LC9 and liver fibrosis in MASLD using vibration‐controlled transient elastography (VCTE); (b) assess the relationship between LC9 and long‐term mortality; and (c) examine whether systemic inflammation mediates these associations. To the best of our knowledge, no prior study has investigated LC9 in the context of MASLD, and the mediating role of SII/PIV in the LC9‐mortality relationship has not been explored.

## 2. Methods

### 2.1. Data Source and Study Individuals

Data for this study were sourced from the NHANES covering the years 2005–2018. The NHANES, a continuous program managed by the National Center for Health Statistics (NCHS) under the Centers for Disease Control and Prevention (CDC), employs a sophisticated, multistage, stratified, and clustered sampling strategy to gather a sample that reflects the U.S. population [13]. The study protocol received approval from the Institutional Review Board at the CDC, and all participants provided informed consent.

The study included two analyses: a cross‐sectional analysis for the association between LC9 and liver fibrosis (using 2017–2018 NHANES data) and a prospective longitudinal cohort analysis for the association between LC9 and all‐cause mortality (using 2005–2016 NHANES data with follow‐up through December 31, 2019).

For the 2005–2018 NHANES cycles, we included participants aged 20 years or older who completed consecutive study phases. Participants were excluded if they (1) had a positive test result for hepatitis B surface antigen or hepatitis C antibody in their serum; (2) consumed a significant amount of alcohol, defined as 20 g or more per day for men and 10 g or more per day for women; and (3) lacked complete data necessary for the diagnosis of MASLD [14–16]. A detailed flowchart illustrating the inclusion and exclusion process is presented in Figure S1.

### 2.2. Diagnosis of MASLD

MASLD refers to patients with steatotic liver disease (SLD) who also have at least one cardiometabolic risk factor while excluding other causes of chronic liver disease. In this study, the diagnosis of MASLD is based on previous research and guidelines [2, 15].

Although liver biopsy is recognized as the gold standard for diagnosing SLD, its invasive nature limits its use. As a result, noninvasive diagnostic methods are commonly used in large sample surveys [17]. Common alternatives include blood‐based biomarkers or liver ultrasound. In this study, we used two distinct approaches to diagnose MASLD based on data from different NHANES cycles.

During the 2005–2016 NHANES cycle, liver ultrasound data were not available, so we employed the widely used US Fatty Liver Index (US‐FLI) for the diagnosis of fatty liver. In the 2017–2018 cycle, VCTE was used to assess both liver fat infiltration and liver stiffness.

The US‐FLI is a practical and widely used tool in epidemiological studies, originally developed using NHANES III data and validated against ultrasound‐confirmed hepatic steatosis [7, 15, 16]. In this study, participants were considered to have SLD if their US‐FLI was 30 or higher in the absence of excessive alcohol or other known causes of chronic liver disease. The detailed methods for calculating the FLI are documented in the literature [7, 15, 16].

VCTE has been validated in previous studies as an effective method for evaluating liver fibrosis in individuals with NAFLD. The procedures and methodologies of VCTE have been described in prior publications [7]. For this study, we included only those participants who completed a VCTE assessment during the 2017–2018 NHANES cycle. In our analysis, we retrieved liver stiffness measurements (LSMs) and controlled attenuation parameter (CAP) values from the VCTE dataset. Participants with CAP values of 274 dB/m or higher were identified as having SLD [17]. Significant fibrosis was defined as LSM ≥8.0 kPa, a threshold widely validated for detecting fibrosis stage ≥2 in MASLD/NAFLD, with reference to the EASL Clinical Practice Guidelines on noninvasive tests for the evaluation of liver disease severity [17, 18].

### 2.3. Assessment of Life Crucial 9

In general, the LC9 index is derived from the LC8 index combined with scores related to depression. The LE8 score comprises four health behaviors—diet, physical activity, nicotine exposure, and sleep duration—and four health indicators: body mass index (BMI), non‐high‐density lipoprotein cholesterol (non‐HDL‐C), blood glucose, and blood pressure [6]. The calculation method for the LE8 score has been detailed in previous studies [6, 19–21]. In brief, a professional panel used the modified Delphi method to assign scores for each of the eight metrics, ranging from 0 to 100, based on the health outcomes and associated risks. The overall LE8 score was derived by averaging the scores of the eight individual metrics.

The 2015 Healthy Eating Index (HEI) was used to assess diet quality. Dietary intake was gathered through two 24‐h dietary recalls and then integrated with the U.S. Department of Agriculture’s food pattern data to determine the HEI scores [22]. Data regarding physical activity, exposure to nicotine, sleep duration, history of diabetes, and medication usage were collected via standardized questionnaires completed by participants. Blood samples were analyzed in a centralized laboratory to measure lipid levels, fasting blood glucose, and glycated hemoglobin (HbA1c).

Depressive symptoms were assessed using the Patient Health Questionnaire‐9 (PHQ‐9), a validated tool for depression screening [4, 23]. The PHQ‐9 score ranges from 0 to 27. To integrate it with the eight LE8 metrics (each scored 0–100), we linearly transformed the PHQ‐9 score to a 0–100 scale using the formula: Depression score (0–100) = (PHQ‐9 raw score/27) × 100, with higher scores indicating worse mental health (lower cardiovascular health contribution). The final LC9 score was then calculated as the unweighted average of the eight LE8 metrics and the transformed depression score, following the method of Ge et al. [4].

### 2.4. Outcome

This study has two primary objectives: first, to examine the association between the LC9 score and liver stiffness in the MASLD population and second, to evaluate whether the LC9 score provides a protective effect on all‐cause mortality in individuals with MASLD. Death records were obtained from the National Death Index through death certificates, with follow‐up data extending up to December 31, 2019 [4].

### 2.5. Other Variables and Inflammatory Indicators

This study used the NHANES database to conduct in‐depth analyses of demographic characteristics and laboratory parameters. Demographic variables included age, gender, ethnicity, marital status, poverty‐to‐income ratio (PIR), and BMI. The smoking status was classified based on self‐reported data.

Medical histories of conditions such as diabetes and hypertension were identified through self‐reports and laboratory tests. Laboratory parameters included measurements of HbA1c, blood glucose, triglyceride, total cholesterol, HDL, LDL, alanine aminotransferase (ALT), and aspartate aminotransferase (AST). The PHQ9 score and HEI were also included. Detailed protocols for sample collection, processing, quality control, and monitoring are documented in the literature [24, 25].

In this study, the inflammatory indicators included SII and PIV [25, 26]. SII and PIV were defined as follows: SII = peripheral platelet counts × neutrophil counts/lymphocyte counts and PIV = (platelet count × neutrophil count × monocyte count)/lymphocyte count.

### 2.6. Statistical Analysis

All analyses accounted for the complex multistage survey design of the NHANES. We applied the 7‐cycle interview examination weights in all descriptive, linear, logistic, and Cox regression models, as recommended by the NCHS guidelines. We described the baseline characteristics of participants with mean ± standard error (SE) for numerical data and percentages for data that fall into categories. In this present study, we used the data from 2005 to 2016 to explore the relationship between the LC9 score and the all‐cause mortality of MASLD patients and explored the relationship between the LC9 score and liver stiffness within the 2017–2018 cycle. We divided the participants into four groups according to the LC9 score quartiles. The characteristics were compared using the analysis of variance or the Kruskal–Wallis test for continuous variables and the chi‐square test for categorical variables. Missing data for covariates (including PIR, HbA1c, ALT, AST, and PHQ‐9) were handled by complete‐case analysis, as the proportion of missingness was less than 5% for each variable. No imputation was performed. Quartiles were chosen to ensure an adequate sample size in each group and to allow for nonlinear trend assessment, as no clinically established cutoffs for LC9 currently exist in the MASLD population.

The correlation between the LC9 score and LSM values was examined using three weighted multivariable linear regression models during the 2017–2018 cycle. The LC9 score was analyzed as a continuous variable ranging from 0 to 100. In linear regression models, the *β* coefficient represents the change in LSM (kPa) per 1‐unit increase in the LC9 score. In logistic and Cox models, OR and HR represent the change per 1‐unit increase in the LC9 score. Covariates were selected a priori based on prior literature and clinical plausibility, including factors known to influence MASLD progression, liver fibrosis, and mortality. In Model 1, no variables were adjusted. In Model 2, adjustments were made for sex, age, ethnicity, education level, and marital status. In Model 3, adjustments were made for sex, age, ethnicity, education level, marital status, smoking status, HbA1c, ALT, AST, hypertension, and diabetes mellitus. We then defined significant fibrosis as an LSM value greater than or equal to 8.0 kPa. The correlation between the LC9 score and liver fibrosis was also examined with three weighted multivariable logistic regression models. We also performed RCS analysis to explore the possibility of nonlinear relationships between LC9 and the presence of liver fibrosis. Multicollinearity among covariates was assessed using variance inflation factor (VIF). All variables included in the final models had VIF <5, indicating no significant collinearity.

We next examined the association between LC9 and mortality in the MASLD population to further ascertain the prognostic value of LC9 in the cycle of 2005–2016. We also conducted weighted multivariate Cox models to examine the associations between LC9 score quartile and survival status. Moreover, we also used RCS analysis to investigate potential nonlinear associations between LC9 and the survival status. The proportional hazards assumption for Cox regression models was tested using Schoenfeld residuals. No significant violations were detected (global test *p*  > 0.05).

Additionally, multivariate linear and Cox regression were performed on the associations between inflammatory indicators with LC9 and all‐cause mortality, respectively. The possible mediating role of systemic inflammation in the relationship between the LC9 score and mortality was assessed using a mediation analysis model. We used the R package mediation to assess the mediating effect, applying 5000 bootstrap resamples as a reliable technique for calculating the bias‐corrected confidence intervals in our analysis. All mediation analyses were based on the Cox model 3. Our results include the indirect effect sizes (βindirect), direct effect sizes (βdirect), total effect sizes (βtotal), the proportion mediated (PM), and the corresponding *p*‐values.

We examined the subgroup interactions of age (as a categorial variable), sex, ethnicity, marital status, and PIR in the association of the LC9 score with mortality by fitting Cox models.

A two‐sided *p*  < 0.05 was considered statistically significant. Analyses were conducted with R software, version 4.1.1 (R Core Team, Vienna, Austria). The following R packages were used: survey (v4.1.1) for weighted analyses, rms (v6.3‐0) for RCS, mediation (v4.5.0) for mediation analysis, and survival (v3.3‐1) for Cox models.

## 3. Results

### 3.1. Baseline Characteristics of the Study Population

After applying the inclusion and exclusion criteria, we included 2524 MASLD participants from the 2005–2016 NHANES cycles and 1277 MASLD participants from the 2017–2018 cycle. The specific inclusion and exclusion processes are illustrated in Figure S1.

In the 2005–2016 cycle, the average age of participants was 55.97 years, with males accounting for 55.83% of the total population. The majority of participants were Caucasian. The overall mortality rate in this cohort was approximately 14%. The participants were stratified into four groups based on the quartiles of the LC9 score. Mortality decreased across increasing LC9 quartiles. Significant differences in demographic characteristics, such as gender, age, and race, were observed among the quartiles. Additionally, laboratory parameters, including HbA1c, blood glucose, LDL, HDL, albumin, and total cholesterol, varied significantly across the quartiles. Detailed data are presented in Table 1.

**Table 1:** Baseline characteristics of MASLD participants from NHANES 2005–2016 stratified by LC9 score quartiles.

Variable	Total	Q1	Q2	Q3	Q4	*p*‐Value
Age (year)	55.97 (0.40)	57.77 (0.72)	56.79 (0.78)	55.05 (0.84)	54.75 (0.70)	0.01
Sex (%)						<0.0001
Female	44.17	53.87	47.12	41.77	36.22	—
Male	55.83	46.13	52.88	58.23	63.78	—
Ethnicity (%)						<0.001
Mexican American	9.2	7.07	8.98	8.68	11.64	—
Non‐Hispanic black	5.6	8.11	7.93	4.97	2.22	—
Non‐Hispanic white	75.32	74.89	73.98	76.81	75.31	—
Other Hispanic	4.35	4.85	4.18	4.45	3.98	—
Other race	5.53	5.09	4.93	5.09	6.86	—
Marital status (%)						0.004
Not married nor living with a partner	30.59	37.39	32.89	27.99	25.75	—
Married or living with a partner	69.40	62.61	67.11	72.01	74.25	—
Poverty to income ratio	2.95 (0.06)	2.45 (0.10)	2.72 (0.09)	3.16 (0.10)	3.33 (0.09)	<0.0001
BMI	34.41 (0.19)	37.37 (0.33)	35.28 (0.34)	34.00 (0.32)	31.70 (0.33)	<0.0001
Glucose (mg/dL)	122.67 (1.13)	145.25 (3.02)	123.82 (1.72)	117.99 (2.24)	108.05 (0.91)	<0.0001
HbA1c	6.09 (0.03)	6.82 (0.09)	6.18 (0.06)	5.92 (0.05)	5.61 (0.03)	<0.0001
Triglyceride (mmol/L)	1.96 (0.04)	2.36 (0.09)	1.99 (0.06)	1.92 (0.07)	1.65 (0.05)	<0.0001
Total cholesterol (mmol/L)	4.98 (0.03)	5.31 (0.06)	5.12 (0.07)	4.84 (0.06)	4.74 (0.05)	<0.0001
HDL (mmol/L)	1.19 (0.01)	1.14 (0.02)	1.21 (0.01)	1.17 (0.02)	1.23 (0.01)	<0.001
LDL (mmol/L)	2.92 (0.03)	3.14 (0.05)	3.03 (0.06)	2.82 (0.06)	2.78 (0.05)	<0.0001
Albumin (g/L)	41.95 (0.09)	40.84 (0.16)	41.87 (0.20)	42.15 (0.15)	42.71 (0.14)	<0.0001
ALT (U/L)	29.75 (0.41)	28.72 (1.00)	29.45 (0.93)	30.90 (0.78)	29.67 (0.78)	0.33
AST (U/L)	26.75 (0.34)	26.57 (0.96)	26.78 (0.50)	27.04 (0.58)	26.57 (0.41)	0.9
SII	573.16 (8.23)	608.47 (16.05)	593.70 (18.32)	583.34 (17.97)	517.08 (13.99)	<0.001
PIV	335.10 (6.36)	356.96 (13.13)	352.12 (14.46)	343.54 (16.13)	294.66 (10.42)	<0.001
PHQ9 score	3.25 (0.12)	5.93 (0.38)	3.57 (0.25)	2.45 (0.15)	1.62 (0.11)	<0.0001
HEI	49.26 (0.34)	43.52 (0.56)	46.75 (0.59)	49.85 (0.77)	55.47 (0.67)	<0.001
DM (%)	36.17	58.27	44.62	31.76	15.49	<0.0001
Hypertension (%)	61.09	79.03	66.5	59.78	43.22	<0.0001
Mortality rate (%)	14.21	19.87	17.14	13.77	7.57	<0.0001

*Note:* Data are expressed as weighted proportions for categorical variables and as weighted means (SE) for continuous variables. Linear regression and Rao–Scott chi‐square test were used to compare groups.

Abbreviations: ALT, alanine aminotransferase; AST, aspartate aminotransferase; BMI, body mass index; DM, diabetes mellitus; HbA1c, glycated hemoglobin; HDL, high‐density lipoprotein; HEI, Healthy Eating Index; LC9, Life’s Crucial 9; LDL, low‐density lipoprotein; MASLD, metabolic dysfunction‐associated steatotic liver disease; PHQ9, Patient Health Questionnaire‐9; PIV, pan‐immune‐inflammation value; SII, systemic immune‐inflammation index.

During the 2017–2018 period, our research was concentrated on examining the association between the LC9 score and liver fibrosis. A total of 1277 participants meeting the inclusion and exclusion criteria were analyzed. The participants were again grouped into quartiles based on the LC9 score. Significant differences in liver fibrosis prevalence were noted among the quartiles, with the prevalence and LSM values both decreasing as the LC9 score increased. Further details are presented in Table S1.

### 3.2. Association Between LC9 Score and Liver Fibrosis

Because VCTE more accurately assesses fibrosis, we used data from the 2017–2018 cycle to explore the relationship between the LC9 score and liver fibrosis. We performed multivariate linear regression analyses between the LC9 score and the LSM value, establishing three models (Table 2). When analyzing the LC9 score as a continuous variable, we found in the fully adjusted Model 3 that the LC9 score was negatively correlated with the LSM value (Model 3: *β* = −0.07,) (Table 2).

**Table 2:** The associations of LC9 index with liver stiffness measurement (kpa) in individuals with MASLD.

Variables	Model 1		Model 2		Model 3	
	*β* (95% CI)	*p*‐Value	*β* (95% CI)	*p*‐Value	*β* (95% CI)	*p*‐Value
LC9						
Continuous variable	−0.06 (−0.10, −0.03)	0.001	−0.07 (−0.11, −0.03)	0.03	−0.07 (−0.12, −0.01)	0.03
Quartiles						
Q1	Ref	—	Ref	—	Ref	—
Q2	−1.54 (−2.89, −0.19)	0.03	−1.51 (−3.17, 0.15)	0.06	−1.14 (−3.22, 0.94)	0.21
Q3	−1.45 (−2.90, −0.01)	0.04	−1.58 (−3.19, −0.02)	0.04	−1.05 (−3.13, 1.03)	0.18
Q4	−2.46 (−3.74, −1.18)	0.001	2.61 (−4.29, −0.93)	0.01	−2.34 (−4.75, −0.01)	0.04
*p* for trend	<0.001	—	<0.001	—	<0.001	—

*Note:* linear regression analysis: Model 1: no adjusted. Model 2: adjusted for covariates including sex, age, ethnicity, education level, and marital status. Model 3: adjusted for covariates including sex, age, ethnicity, education level, marital status, smoking status, HbA1c, ALT, AST, hypertension, and diabetes mellitus.

**Figure 1:**
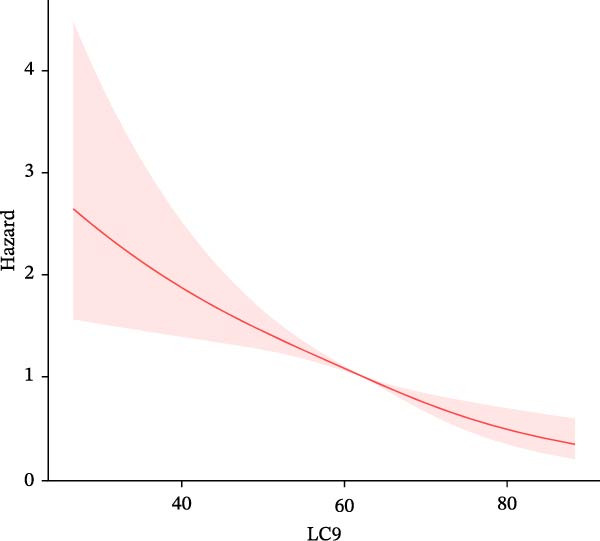
The analysis employs restricted cubic splines (RCS) to examine the multivariate‐adjusted relationship between the LC9 score and liver fibrosis. The solid red lines represent the central estimates of the association, while the red‐shaded areas denote the corresponding 95% confidence intervals.

Similar results were observed in logistic regression analyses, defining liver fibrosis as an LSM value ≥8 kPa. The adjustment variables remained constant. The findings showed that with each 1‐unit rise in the LC9 score, the risk of fibrosis dropped by 5% (Model 3: OR = 0.95). Furthermore, when stratifying the LC9 score into quartiles, participants in the Q4 group exhibited the lowest risk of fibrosis compared to other subgroups (Model 3: OR = 0.21,). Detailed results are presented in Table S2. Additional RCS analysis confirmed an approximately linear relationship between LC9 score and risk of fibrosis (Figure 1). In the stratified analysis, the link between the LC9 score and fibrosis was found to be consistently present, as illustrated in Figure S2.

### 3.3. Association Between LC9 Score and All‐Cause Mortality

**Figure 2:**
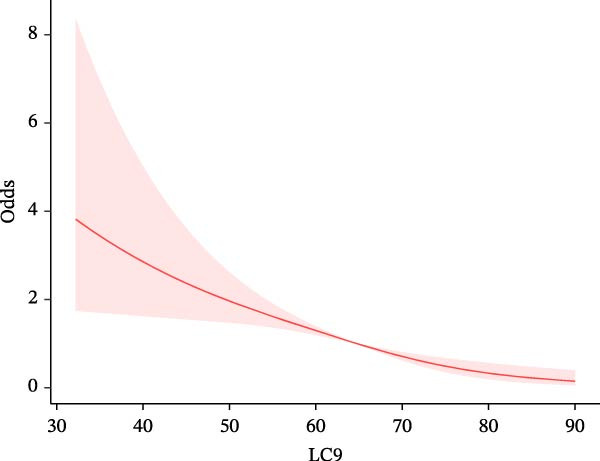
The analysis employs restricted cubic splines (RCS) to examine the multivariate‐adjusted relationship between the LC9 score and all‐cause mortality. The solid red lines represent the central estimates of the association, while the red‐shaded areas denote the corresponding 95% confidence intervals.

A median follow‐up time of 98 months was obtained. Multivariate Cox regression analysis further revealed that for each one unit increase in LC9 score, the long‐term mortality rate decreased by 3% (HR = 0.97) (Table 3). In Model 3, the mortality rate in the highest group (Q4) was 0.42 (95%CI: 0.27–0.66) times that of the lowest intake group (Q1), indicating that the LC9 score is beneficial for the long‐term prognosis of MASLD individuals (Table 3). Additional RCS analysis confirmed an approximately linear relationship between LC9 score and improved long‐term survival (Figure 2). Subgroup analysis showed that this protective effect remained consistent in different subgroups (Figure S3).

**Table 3:** The associations of LC9 with all‐cause mortality in individuals with MASLD.

Variables	Deaths/participants	Model 1		Model 2		Model 3	
		HR (95% CI)	*p*‐Value	HR (95% CI)	*p*‐Value	HR (95% CI)	*p*‐Value
LC9							
Continuous variable	—	0.97 (0.96, 0.98)	<0.0001	0.97 (0.96, 0.98)	<0.0001	0.97 (0.96, 0.98)	<0.0001
Quartiles							
Q1	126/631	Ref	—	Ref	—	Ref	—
Q2	109/631	0.86 (0.66, 1.14)	0.15	0.81 (0.61, 1.07)	0.21	0.93 (0.67, 1.28)	0.18
Q3	86/631	0.67 (0.52, 0.88)	0.001	0.67 (0.53, 0.86)	0.003	0.75 (0.58, 0.97)	0.03
Q4	46/631	0.38 (0.25, 0.58)	<0.001	0.34 (0.22, 0.52)	<0.001	0.42 (0.27, 0.66)	<0.001
*p* for trend	—	<0.001	—	<0.001	—	<0.001	—

*Note:* Cox regression analysis: Model 1: no adjusted. Model 2: adjusted for covariates including sex, age, ethnicity, education level, and marital status. Model 3: adjusted for covariates including sex, age, ethnicity, education level, marital status, smoking status, HbA1c, ALT, AST, LSM, hypertension, and diabetes mellitus.

### 3.4. Associations of Inflammation With LC9 Score and All‐cause Mortality

Table 4 shows the results of the analysis concerning the LC9 score and its association with the inflammation markers. After accounting for all potential confounders, the LC9 score exhibited a negative association with both the SII (*β* = −2.61,) and the PIV (*β* = −1.96, *p*  = 0.004). Table S3 presents the results from Cox regression models exploring the relationship between inflammation‐related indicators and all‐cause mortality. Both SII and PIV were positively associated with an increased mortality risk.

**Table 4:** The associations of LC9 with systemic inflammation index and pan‐immune‐inflammation value.

Models	*β* value	95% CI	*p*‐Value
SII			
Model 1	−2.58	(−3.92, −1.25)	0.001
Model 2	−2.53	(−4.01, −1.05)	0.001
Model 3	−2.61	(−3.94, −1.27)	0.002
PIV			
Model 1	−2	(−3.08, −0.91)	<0.001
Model 2	−1.98	(−3.31, −0.64)	0.004
Model 3	−1.96	(−3.27, −0.99)	0.004

*Note:* linear regression analysis: Model 1: no adjusted. Model 2: adjusted for covariates including sex, age, ethnicity, education level, and marital status. Model 3: adjusted for covariates including sex, age, ethnicity, education level, marital status, smoking status, HbA1c, ALT, AST, hypertension, and diabetes mellitus.

### 3.5. Mediating Role of Inflammation‐Related Indicators

**Figure 3:**
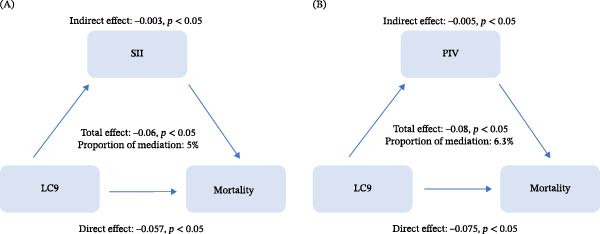
Analysis of the mediation by SII (A) and PIV (B) of the associations of LC9 with all‐cause mortality.

To further investigate the role of systemic inflammation in the improved prognosis associated with the LC9 score, we conducted mediation analysis. As shown in Figure 3, the SII mediated 5% of the relationship between the LC9 score and all‐cause mortality. For the analysis involving the PIV, the mediation proportion was 6.3%, as detailed in Table S4.

## 4. Discussion

In our research, we enrolled a total of 3801 participants with MASLD, maintaining a roughly equal male‐to‐female ratio. Higher LC9 scores were associated with lower LSM values. Specifically, for each 1‐unit increment in the LC9 score, there was a 5% decrease in the risk of significant fibrosis. Additionally, higher LC9 scores were associated with improved long‐term survival, with a 3% decrease in all‐cause mortality for each unit increase in LC9. These findings suggest that LC9, a multidimensional score integrating cardiovascular and metabolic health, may help identify patients at higher risk for liver fibrosis and poor long‐term outcomes in MASLD patients.

Previous studies have indicated that a higher LE8 score is associated with a lower prevalence of NAFLD [27, 28]. While LE8 has been shown to be protective against MASLD prevalence and fibrosis, our study demonstrates that LC9—which adds a mental health component—is also inversely associated with liver fibrosis. The inclusion of depression is clinically relevant, as depression is highly prevalent in MASLD patients, likely driven by shared mechanisms including chronic inflammation, metabolic disturbances, and psychosocial stress [29, 30]. Cai et al. [31] reported a positive relationship between PHQ‐9 scores and MASLD prevalence. However, they did not find that an increase in PHQ‐9 scores significantly affects the fibrosis risk. LC9 provides a broader assessment by integrating lifestyle habits, metabolic characteristics, blood tests, and mental health. This suggests that depression may influence the development of steatosis rather than its progression to fibrosis. By integrating lifestyle habits, metabolic metrics, and mental health into a single score, LC9 offers a more holistic risk assessment than LE8 alone. The protective effect we observed for LC9 against liver fibrosis, despite the lack of a direct fibrosis signal from PHQ‐9 alone, suggests multidimensional risk scores that capture synergistic effects across domains.

Additionally, patients with metabolic disorders often experience chronic low‐grade inflammation, which can worsen insulin resistance, promote liver fibrosis, and elevate mortality risk [32–34]. Our findings are consistent with previous studies, showing that systemic inflammation markers such as SII and PIV are positively associated with long‐term mortality in MASLD patients. Importantly, we found that a higher LC9 score is associated with lower SII and PIV values. Mediation analysis further revealed that a portion of LC9’s protective effect on prognosis is mediated through its ability to reduce chronic systemic inflammation. These findings highlight the potential importance of targeting chronic low‐grade inflammation in MASLD management, with LC9 serving as a useful tool for promoting healthy lifestyles that reduce inflammation and improve patient outcomes.

Previous studies have demonstrated that the LE8 score correlates with decreased rates of mortality. Two large cohort studies, one utilizing data from the UK Biobank and the other from NHANES, have both confirmed the protective role of LE8 in reducing mortality rates in the general population [35, 36]. The LC9 score, which incorporates a depression score into the LE8 framework, has also been shown to improve prognosis in recent studies using the NHANES database. However, its impact on long‐term outcomes in specific populations, such as those with MASLD, remains unclear. To address this gap, we conducted a prospective cohort study using NHANES data. Our study results indicate that the LC9 score is considerably correlated with a decrease in mortality rates among patients with MASLD, supporting the conclusions of previous research and extending its applicability to this at‐risk population.

This study offers several strengths. First, liver fibrosis was assessed using VCTE, which provides a more accurate and reliable measurement compared to non‐invasive blood‐based markers. Second, the use of the NHANES database enabled a large, nationally representative sample, enhancing the generalizability of our findings. Third, the application of LC9, a novel multidimensional metric of cardiovascular and metabolic health, provides a broader assessment of patient health. To our knowledge, this study is the first to explore the link between the LC9 score and liver fibrosis, along with long‐term outcomes, in patients with MASLD. Furthermore, our findings indicate that LC9’s beneficial effects on prognosis may be partially mediated by reductions in systemic inflammation, as reflected by SII and PIV, highlighting an important mechanistic pathway.

The mediation proportions for SII (5%) and PIV (6.3%) were modest, indicating that systemic inflammation explains only a small fraction of the protective effect of LC9 on mortality. Several factors may account for this. First, LC9 encompasses diverse domains including blood pressure, lipids, glucose, and mental health, which likely influence mortality through multiple pathways beyond inflammation—such as direct cardiovascular protection, improved insulin sensitivity, and reduced oxidative stress. Second, inflammation was measured at a single time point using composite indices derived from complete blood counts; more specific inflammatory markers (e.g., IL‐6 and TNF‐α) might yield different mediation estimates. Third, residual confounding cannot be ruled out. Thus, while inflammation partially mediates the relationship, the majority of LC9’s effect appears to operate through other mechanisms.

One might question whether LC9 is simply a proxy for better metabolic control, given that it includes metrics such as BMI, blood glucose, and lipids. However, several observations suggest otherwise. First, LC9 also incorporates behavioral (diet, physical activity, sleep, and smoking) and mental health (depression) domains, which are not captured by conventional metabolic control measures (e.g., HbA1c alone). Second, in our multivariable models, LC9 remained significantly associated with mortality even after adjusting for individual metabolic parameters, suggesting an independent effect. Third, the partial mediation by inflammation implies that LC9 captures risk beyond what is reflected by standard metabolic markers. Nevertheless, we acknowledge that disentangling the unique contribution of LC9 from its metabolic components is challenging, and future studies using formal decomposition methods may help clarify this issue.

However, it is important to acknowledge the limitations inherent in our study. First, the cross‐sectional methodology employed to investigate the link between the LC9 score and liver fibrosis cannot establish causality. Prospective longitudinal studies are needed to establish causal links. Second, while liver fibrosis was assessed with VCTE in the 2017–2018 cycle, the diagnosis of MASLD in earlier cycles relied on blood‐based criteria due to the unavailability of VCTE data. Nonetheless, these diagnostic approaches are widely validated and accepted. Third, despite extensive adjustment for potential confounders, residual confounding may remain. Fourth, the diagnosis of MASLD relied on different methods across NHANES cycles: US‐FLI (2005–2016) and VCTE (2017–2018). While both are validated noninvasive tools, this heterogeneity may introduce measurement variability. However, this reflects the practical constraints of large‐scale epidemiologic studies, and our analytic approach kept the two components separate. Fifth, while the temporal sequence (LC9 and inflammation measured at baseline and mortality assessed prospectively) supports the directionality of the mediation analysis, causal inference from observational mediation analysis should be made with caution. Future studies should aim to address these limitations and further validate the utility of LC9 in MASLD risk stratification and management.

## 5. Conclusion

This study demonstrates that higher LC9 scores are associated with reduced liver fibrosis and improved survival in MASLD, partially mediated by lower systemic inflammation. Utilizing VCTE and a large representative cohort, we provide the first evidence linking LC9 to MASLD outcomes. LC9 may be useful for risk stratification, emphasizing its potential role in clinical practice and public health.

## Author Contributions

Zhuo Chen, Lin Shi, Junjie Lu, and Jing Su contributed to study concept and design, acquisition of data, analysis and interpretation of data, drafting of the manuscript, critical revision of the manuscript for important intellectual content, and statistical analysis.

## Funding

This study was funded by the Development Fund of the Affiliated Hospital of Xuzhou Medical University (Grant XYFM202339).

## Disclosure

All the authors have read and approved the final manuscript.

## Conflicts of Interest

The authors declare no conflicts of interest.

## Supporting Information

Additional supporting information can be found online in the Supporting Information section.

## Supporting information


**Supporting Information** Table S1. Baseline characteristics of study population from NHANES 2017−2018. Table S2. The associations of LC9 index with liver fibrosis in individuals with MASLD. Table S3. The associations of inflammation‐related indicators and all‐cause mortality. Table S4. Analysis of the mediation by inflammation‐related indicators of the associations of LC9 with all‐cause mortality in individuals with MASLD. Figure S1. The flow chart of our study. Figure 1A shows the screening process of the NHANES 2005–2016 cycle. Figure 1B shows the screening process of the 2017–2018 cycle. Figure S2. Subgroup analysis and interaction of the association between LC9 and liver fibrosis in MASLD. OR, odds ratio. The black rectangles correspond to the central estimates, and the black lines indicate the 95% confidence intervals. Figure S3. Subgroup analysis and interaction of the association between LC9 and all‐cause mortality in MASLD. HR, hazard ratio. The black rectangles correspond to the central estimates, and The black lines indicate the 95% confidence intervals.

## Data Availability

Publicly available datasets were analyzed in this study. This data can be found at https://www.cdc.gov/nchs/nhanes.
