# Supplementary material for: Association of Life’s Crucial 9 Score With Liver Fibrosis and Mortality in U.S. Adults With MASLD: Evidence From NHANES and the Mediating Role of Systemic Inflammation
Source: Mediators Inflamm. 2026 Jun 19;2026:2700500. doi: 10.1155/mi/2700500 (PMC13280809; doi:10.1155/mi/2700500)
Supplement: Supplementary file 1 — Supporting Information Table S1. Baseline characteristics of study population from NHANES 2017−2018. Table S2. The associations of LC9 index with liver fibrosis in individuals with MASLD. Table S3. The associations of inflammation‐related indicators and all‐cause mortality. Table S4. Analysis of the mediation by inflammation‐related indicators of the associations of LC9 with all‐cause mortality in individuals with MASLD. Figure S1. The flow chart of our study. Figure 1A shows the screening process of the NHANES 2005–2016 cycle. Figure 1B shows the screening process of the 2017–2018 cycle. Figure S2. Subgroup analysis and interaction of the association between LC9 and liver fibrosis in MASLD. OR, odds ratio. The black rectangles correspond to the central estimates, and the black lines indicate the 95% confidence intervals. Figure S3. Subgroup analysis and interaction of the association between LC9 and all‐cause mortality in MASLD. HR, hazard ratio. The black rectangles correspond to the central estimates, and The black lines indicate the 95% confidence intervals. [file MI-2026-2700500-s001.docx]

**Supplementary materials**

Supplementary Table 1 Baseline characteristics of study population from NHANES 2017-2018

| variable | total | Q1 | Q2 | Q3 | Q4 | P-value |
| --- | --- | --- | --- | --- | --- | --- |
| Age, year | 55.03(0.54) | 58.15(1.10) | 54.84(0.84) | 53.27(1.13) | 52.71(1.33) | 0.01 |
| Sex, % |  |  |  |  |  | 0.61 |
| Female | 44.85 | 49.87 | 43.88 | 42.06 | 42.24 |  |
| Male | 55.15 | 50.13 | 56.12 | 57.94 | 57.76 |  |
| Ethnicity, % |  |  |  |  |  | < 0.001 |
| Mexican American | 8.18 | 6.67 | 8.26 | 9.18 | 9.08 |  |
| Non-Hispanic Black | 9.91 | 15.25 | 9.26 | 8.33 | 4.19 |  |
| Non-Hispanic White | 65.62 | 63.75 | 69.01 | 68.57 | 58.18 |  |
| Other Hispanic | 5.27 | 3.41 | 5.66 | 6.2 | 6.37 |  |
| Other Race | 11.01 | 10.93 | 7.81 | 7.72 | 22.17 |  |
| Marital status, % |  |  |  |  |  | 0.06 |
| Not married nor living with a  partner | 27.24 | 35.65 | 22.35 | 23.15 | 27.77 |  |
| Married or living with a partner | 72.67 | 64.35 | 77.65 | 76.85 | 72.23 |  |
| Poverty to income ratio | 3.21(0.10) | 2.63(0.11) | 3.39(0.13) | 3.32(0.14) | 3.73(0.17) | < 0.001 |
| BMI | 32.63(0.36) | 35.82(0.54) | 32.90(0.66) | 30.83(0.40) | 29.47(0.68) | < 0.0001 |
| Glucose, mg/dl | 121.64(3.14) | 144.48(5.34) | 117.35(2.87) | 117.06(5.37) | 104.87(0.93) | < 0.0001 |
| HbA1c | 5.98(0.04) | 6.48(0.06) | 6.03(0.05) | 5.76(0.09) | 5.42(0.04) | < 0.0001 |
| Triglyceride, mmol/L | 1.60(0.09) | 2.09(0.19) | 1.76(0.16) | 1.47(0.11) | 0.88(0.04) | < 0.0001 |
| Total cholesterol, mmol/L | 4.94(0.06) | 5.13(0.10) | 5.13(0.10) | 4.76(0.09) | 4.56(0.09) | 0.004 |
| HDL, mmol/L | 1.26(0.01) | 1.18(0.03) | 1.24(0.02) | 1.26(0.03) | 1.43(0.06) | 0.04 |
| LDL, mmol/L | 2.92(0.08) | 3.10(0.11) | 3.20(0.12) | 2.68(0.14) | 2.59(0.11) | 0.02 |
| ALT, U/L | 24.19(0.40) | 23.79(0.95) | 24.53(1.18) | 24.17(0.89) | 24.32(0.94) | 0.96 |
| AST, U/L | 21.53(0.40) | 21.14(0.86) | 21.30(0.46) | 21.31(0.57) | 22.91(0.94) | 0.19 |
| SII | 518.15(13.58) | 545.21(21.00) | 537.73(23.58) | 510.34(19.13) | 451.25(27.43) | 0.06 |
| PIV | 319.73(10.57) | 363.16(18.99) | 339.32(18.45) | 301.60(15.56) | 242.23(20.81) | 0.01 |
| PHQ9 score | 2.78(0.14) | 4.73(0.32) | 2.64(0.19) | 1.72(0.10) | 1.30(0.14) | <0.001 |
| HEI | 49.06(0.59) | 45.05(1.01) | 45.96(0.92) | 50.79(1.19) | 58.71(1.24) | <0.0001 |
| LSM, kPa | 6.19(0.21) | 7.41(0.57) | 5.87(0.24) | 5.96(0.32) | 4.96(0.18) | 0.002 |
| DM, % | 27.08 | 44.67 | 29.09 | 20.01 | 4.09 | < 0.0001 |
| Hypertension,% | 53.71 | 71.21 | 58.21 | 47.62 | 24.87 | < 0.0001 |
| Liver Fibrosis, % | 13.89 | 23.53 | 12.76 | 10.18 | 4.91 | 0.002 |

Note: Data are expressed as weighted proportions for categorical variables and as weighted means (SE) for continuous variables. Linear regression and Rao-Scott chi-square test were used to compare groups.

Abbreviations: MASLD, metabolic dysfunction-associated steatotic liver disease; LC9, Life's Crucial 9; BMI, body mass index; HbA1c, glycated hemoglobin; HDL, high-density lipoprotein; LDL, low-density lipoprotein; ALT, alanine aminotransferase; AST, aspartate aminotransferase; SII, systemic immune-inflammation index; PIV, pan-immune-inflammation value; PHQ9, Patient Health Questionnaire-9; HEI, Healthy Eating Index; DM, diabetes mellitus.

Supplementary Table 2 The associations of LC9 index with liver fibrosis in individuals with MASLD

|  | Model 1 |  | Model 2 |  | Model 3 |  |
| --- | --- | --- | --- | --- | --- | --- |
|  | OR (95% CI) | P value | OR (95% CI) | P value | OR (95% CI) | P value |
| LC9 |  |  |  |  |  |  |
| Continuous variable | 0.96(0.94,0.97) | <0.0001 | 0.96(0.94,0.97) | 0.001 | 0.95(0.92,0.98) | 0.01 |
| Quartiles |  |  |  |  |  |  |
| Q1 | Ref |  | Ref |  | Ref |  |
| Q2 | 0.48(0.21,1.10) | 0.08 | 0.47(0.15,1.42) | 0.14 | 0.47(0.09,2.43) | 0.21 |
| Q3 | 0.37(0.22,0.61) | 0.001 | 0.37(0.18,0.76) | 0.02 | 0.36(0.13,1.05) | 0.07 |
| Q4 | 0.17(0.10,0.28) | <0.0001 | 0.16(0.07,0.33) | 0.001 | 0.21(0.02,0.69) | 0.04 |
| P for trend | <0.001 |  | <0.001 |  | <0.001 |  |

Note: logistic regression analysis: Model 1: No adjusted. Model 2: Adjusted for covariates including gender, age, ethnicity, education level, and marital status. Model 3: Adjusted for covariates including sex, age, ethnicity, education level, marital status, smoking status, HbA1c, ALT, AST, hypertension, and diabetes mellitus.

Supplementary Table 3 The associations of inflammation-related indicators and all-cause mortality

|  | Model 1 |  | Model 2 |  | Model 3 |  |
| --- | --- | --- | --- | --- | --- | --- |
|  | HR (95%CI) | P value | HR (95%CI) | P value | HR (95%CI) | P value |
| Mortality |  |  |  |  |  |  |
| SII | 1.05 (1.03,1.07) | <0.001 | 1.05 (1.03,1.08) | 0.001 | 1.05 (1.03,1.07) | 0.001 |
| PIV | 1.10 (1.07,1.13) | <0.001 | 1.07 (1.03,1.10) | 0.001 | 1.06 (1.03,1.09) | 0.002 |

Note: Cox regression analysis: Model 1: No adjusted. Model 2: Adjusted for covariates including sex, age, ethnicity, education level, and marital status. Model 3: Adjusted for covariates including sex, age, ethnicity, education level, marital status, smoking status, HbA1c, ALT, AST, LC9 index, hypertension, and diabetes mellitus.

Supplementary Table 4 Analysis of the mediation by inflammation-related indicators of the associations of LC9 with all-cause mortality in individuals with MASLD

|  | Mediation effect (95% CI), P value | | | |
| --- | --- | --- | --- | --- |
|  | Total effect | Indirect effect | Direct effect | Mediation,% |
| All-cause mortality |  |  |  |  |
| SII | -0.06 (-0.11, -0.01) | -0.003 (-0.012, -0.001) | -0.057 (-0.072, -0.011) | 5% |
| PIV | -0.08 (-0.12, -0.02) | -0.005 (-0.015, -0.002) | -0.075 (-0.091, -0.015) | 6.3% |

Adjusted for covariates including sex, age, ethnicity, education level, marital status, smoking status, HbA1c, ALT, AST, LC9 index, hypertension, and diabetes mellitus.

Figure legends


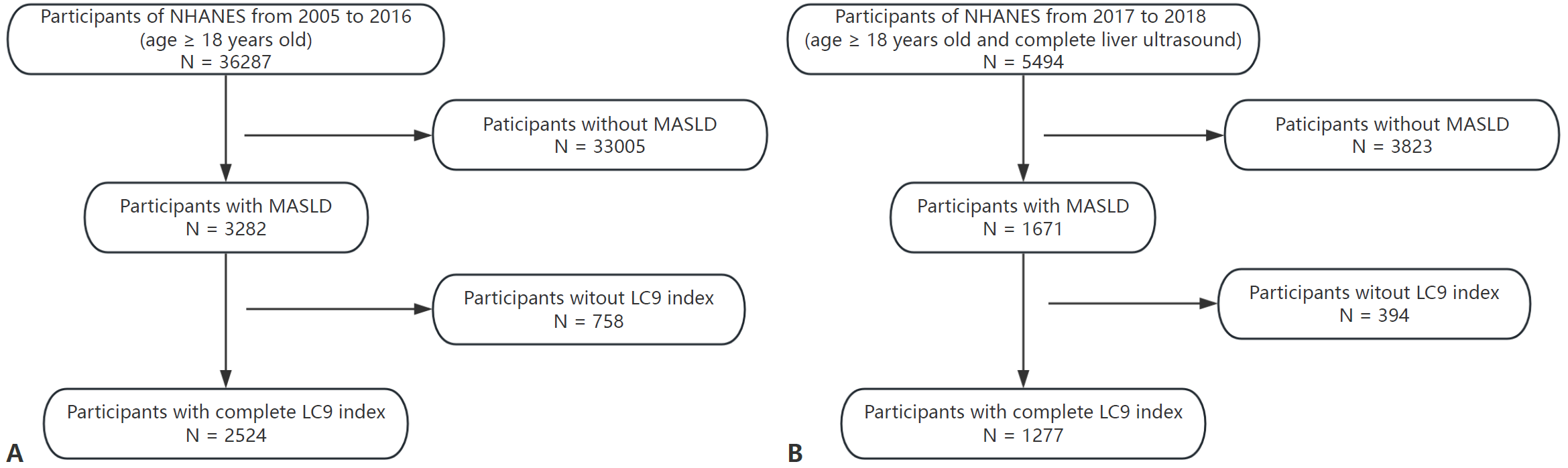


Supplementary Figure 1: The flow chart of our study. Figure 1A shows the screening process of the NHANES 2005 - 2016 cycle. Figure 1B shows the screening process of the 2017 - 2018 cycle.


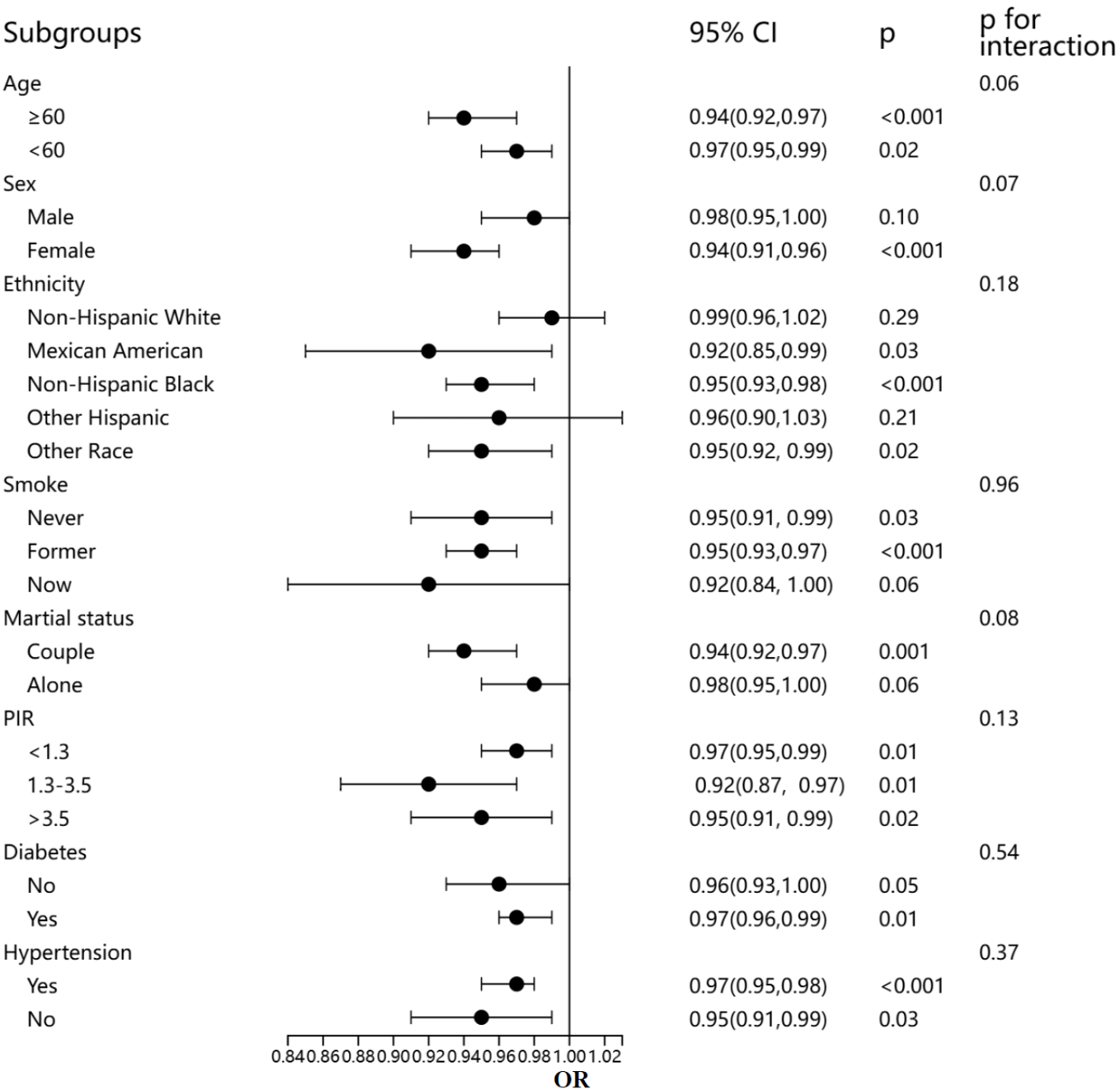


Supplementary Figure 2: Subgroup analysis and interaction of the association between LC9 and liver fibrosis in MASLD. OR, odds ratio. The black rectangles correspond to the central estimates, and The black lines indicate the 95% confidence intervals.


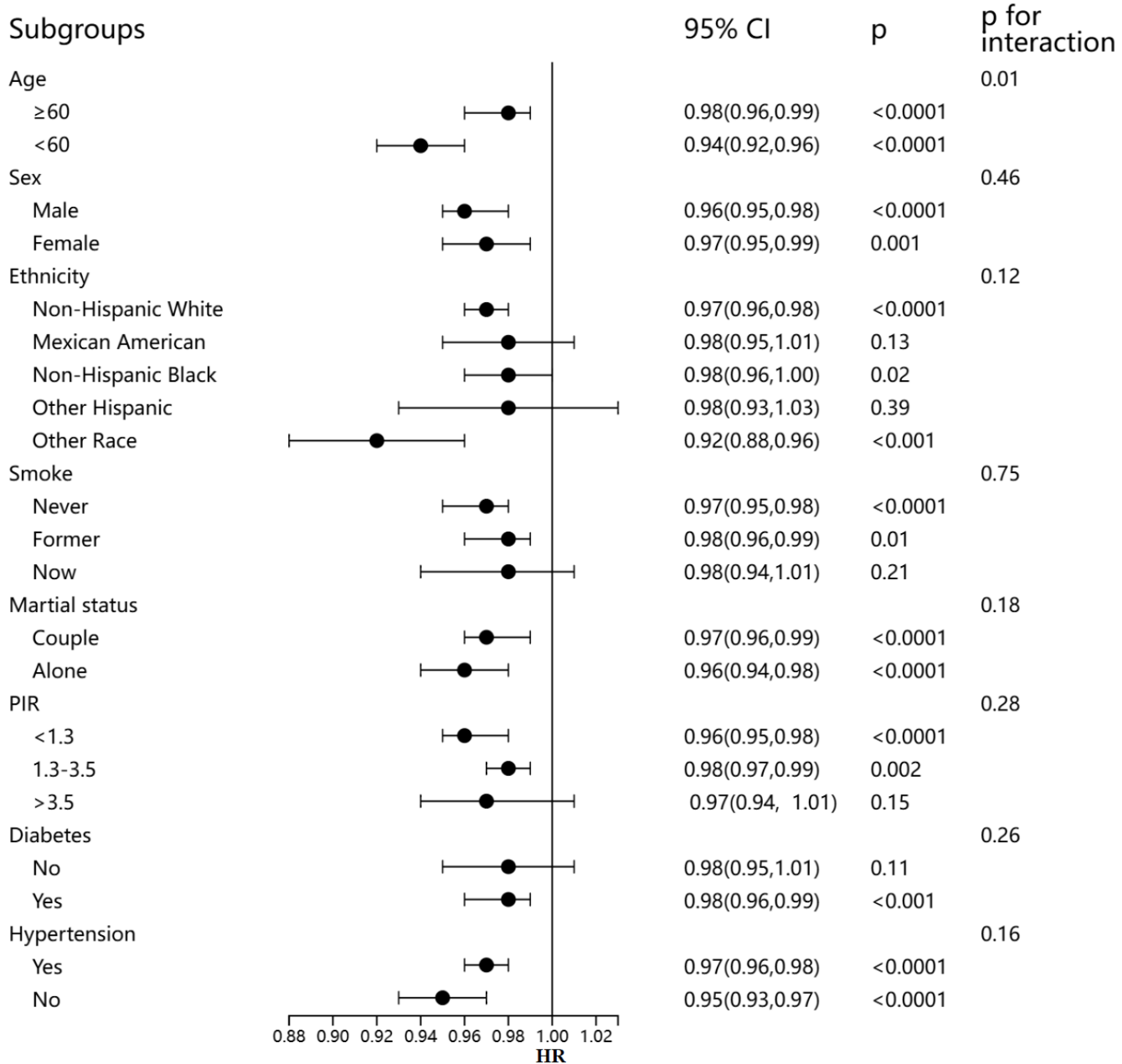


Supplementary Figure 3: Subgroup analysis and interaction of the association between LC9 and all-cause mortality in MASLD. HR, hazard ratio. The black rectangles correspond to the central estimates, and The black lines indicate the 95% confidence intervals.
